# Different dosage regimens of Eptinezumab for the treatment of migraine: a meta-analysis from randomized controlled trials

**DOI:** 10.1186/s10194-021-01220-y

**Published:** 2021-03-06

**Authors:** Zeya Yan, Tao Xue, Shujun Chen, Xin Wu, Xingyu Yang, Guangjie Liu, Shan Gao, Zhouqing Chen, Zhong Wang

**Affiliations:** 1 Department of Neurosurgery & Brain and Nerve Research Laboratory, The First Affiliated Hospital of Soochow University, 188 Shizi Street, Suzhou, 215006 Jiangsu Province China; 2 Department of Neurology, The First Affiliated Hospital of Soochow University, Suzhou, 215006 Jiangsu Province China; 3 Department of Neurosurgery, The People’s Hospital of SND, Suzhou, 215129 Jiangsu Province China

**Keywords:** Eptinezumab, Migraine, Dosage regimen, Meta-analysis

## Abstract

**Background:**

Migraine is one of the most common neurological diseases around the world and calcitonin gene-related peptide (CGRP) plays an important role in its pathophysiology. Therefore, in the present study, we evaluated the efficacy of monoclonal antibodies blocking the CGRP ligand or receptor in episodic and chronic migraine.

**Objective:**

The objective of our study is implementing a meta-analysis to systematically evaluate the efficacy and safety of eptinezumab for the treatment of migraine compared with placebo.

**Method:**

We searched the Medline, Embase, Cochrane Library and Clinicaltrials.gov for randomized controlled trials (RCTs) which were performed to evaluate eptinezumab versus placebo for migraine up to September 2020. The data was assessed by Review Manager 5.3 software. The risk ratio (RR) and standard mean difference (SMD) were analyzed using dichotomous outcomes and continuous outcomes respectively with a random effect model.

**Result:**

We collected 2739 patients from 4 RCTs: the primary endpoint of efficacy was the change from baseline to week 12 in mean monthly migraine days (MMDs). We found that eptinezumab (30 mg, 100 mg, 300 mg) led to a significant reduction in MMDs (*P* = 0.0001,*P* < 0.00001, *P* < 0.00001) during 12 weeks compared with placebo, especially with 300 mg. For the safety, we compared and concluded the treatment emergent adverse events (TEAEs) of the 4 RCTs. This indicated no evident statistical difference between eptinezumab and placebo.

**Conclusions:**

In the present study, we found that eptinezumab is safe and has significant efficacy in the treatment of migraine, especially the dose of 300 mg.

**Supplementary Information:**

The online version contains supplementary material available at 10.1186/s10194-021-01220-y.

## Introduction

Migraine is one of the most prevalent disorders in neurologic field which can be characterized by attacks of headache, hypersensitivity to sound and light stimulation, nausea and vomiting [1]. Commonly, migraine can be divided into episodic migraine and chronic migraine. Episodic migraine is defined as headache which occurs on fewer than 15 days per month, while chronic migraine on 15 or more days per month for at least 3 months or having the features of migraine at least 8 days per month [2]. Presently, migraine has disturbed the peaceful life of more than 16% global population [3, 4]. Until now, the primary goals of migraine treatment include reducing headache frequency, relieving pain and restoring function. Broadly, improvement of physical and psychological health and some other therapies such as pharmacotherapy which will be mainly discussed in the following part are included in treatment strategies [5].

Patients with a few migraine attacks per month can be managed with effective acute therapy. For some patients, nonpharmacologic intervention such as taking a break is enough to relieve the symptom of migraine, while the others need some nonspecific analgesics such as NSAIDs [6]. If the patients are insensitive to nonspecific analgesics, migraine-specific treatment such as triptans will be a better choice. Triptans, the classical drugs of migraine which were widely used over past decades, include sumatriptan, eletriptan, rizatriptan, almotriptan, zolmitriptan, naratriptan, frovatriptan and so on [7]. Moreover, ergots also can be used for the treatment of migraine, but those drugs have not been recommended because of the poor efficacy and adverse reactions compared with triptans. Generally, these drugs are effective and safe in appropriate dose but they occasionally cause an overuse of acute medication in some patients. In addition, drugs for acute treatment have not exhibited obvious advantages on the preventive treatment for migraine. Therefore, further studies are needed on the prophylactic medication, such as monoclonal antibodies against calcitonin gene-related peptide (CGRP) or its receptor, antihypertensives, anticonvulsants, antidepressants, botulinum toxin (the latter only for chronic migraine) [8, 9].

The release of CGRP plays an important role in migraine pathophysiology, which has been observed after the migraine attack [10, 11]. Over the past few years, the efficacy of monoclonal antibodies blocking the CGRP ligand or receptor including galcanezunab, fremanezumab, erenumab and ubrogepant have been demonstrated in both episodic and chronic migraine [12]. Eptinezumab (ALD 403), a new monoclonal antibody that selectively inhibits both α-CGRP and β-CGRP, was available in the market since February 2019. However, there were no systematic review or meta-analysis comprehensively evaluating the efficacy and safety of eptinezumab in the treatment of migraine [13–15].

Therefore, in the present study, we performed a meta-analysis to discuss different dosage regimen of eptinezumab for the treatment of migraine. In the previous clinical trials, eptinezumab had exhibited flexible dosing regimens (10 mg, 30 mg, 100 mg, 300 mg, 1000 mg). During our study, we combined different doses of eptinezumab to analyze the efficacy and safety for the therapy of episodic and chronic migraine [16–19].

## Methods

### Study protocol

Before we started the project, we drafted a research protocol by following the Cochrane Collaboration format [20]. The meta-analysis was not registered.

### Search strategy

Original researches in the MEDLINE, Embase, Cochrane Library and Clinicaltrials.gov were searched using the following terms: [(“eptinezumab and migraine”) (“ALD403 and migraine”)] until September 2020. Moreover, to make sure all relevant studies have been included, we screened reference lists of relevant articles manually.

### Study selection

Studies were included as follows: (1) study type was randomized clinical trials; (2) enrolled participants diagnosed with migraine; (3) study used eptinezumab as intervention; (4) study period was over 12 months; (5) participants were over 18 years old. Studies were excluded as follows: (1) types of study: retrospective studies, cohort studies, case reviews and case reports; (2) control: active control (i.e. that a known, effective treatment as opposed to a placebo is compared with an experimental treatment).

### Data extraction

All the data were extracted independently by 2 investigators (ZYY and TX) and any disagreements were settled through discussion. After several selections and assessments, the basic information of the included trails (first author, publication, country, centers, and treatment groups), patient characteristics (Age range, mean age and gender), study period and outcome events were used to extract the data (Table 1).

**Table 1:** Characteristics of the Included Studies and Outcome Events

Study	Countries	Centers	Publication	Type of migraine	Treatment group, (No. of participants)	Total number	Age range	Female (%)	Mean age ± SD (year)	Study period	Outcome Events
Dodick et al. 2014 (NCT01772524)	USA	26	Lancet Neurol	episodic	Eptinezumab1000mg (81) vs. PLA (82)	163	18y-55y	Eptinezumab 1000 mg:83 PLA:80	Eptinezumab 1000 mg:38.6 ± 10.8 PLA:39.0 ± 9.6	12 months	a,b,c,d,e,f,g,h
Dodick et al. 2019 (NCT02275117)	5	92	Cephalalgia	chronic	Eptinezumab300mg (121) vs.100 mg (122) vs. 30 mg (122) vs.10 mg (130) vs. PLA (121)	616	18y-55y	Eptinezumab 300 mg:81 Eptinezumab 100 mg:85 Eptinezumab 30 mg:91 Eptinezumab 10 mg:87 PLA:90	Eptinezumab 300 mg:37.2 ± 10.0 Eptinezumab 100 mg:36.7 ± 9.4 Eptinezumab 30 mg:35.7 ± 9.4 Eptinezumab 10 mg:36.4 ± 10.3 PLA:37.2 ± 9.2	24 months	a,b,c,d,f,g,h,j
Ashina et al. 2020 (NCT02559895)	2	84	Cephalalgia	episodic	Eptinezumab300mg (224) vs.100 mg (223) vs.30 mg (219) vs. PLA (222)	888	18y-75y	Eptinezumab 300 mg:89 Eptinezumab 100 mg:80 Eptinezumab 30 mg:84 PLA:84	Eptinezumab 300 mg:40.2 ± 11.72 Eptinezumab 100 mg:40.0 ± 10.66 Eptinezumab 30 mg:39.1 ± 11.54 PLA:39.9 ± 11.67	26 months	a,b,c,d,g,j
Lipton et al. 2020 (NCT02974153)	13	128	Neurology	chronic	Eptinezumab300mg (350) vs.100 mg (356) vs. PLA (366)	1072	18y-65y	Eptinezumab 300 mg:90 Eptinezumab 100 mg:86 PLA:89	Eptinezumab 300 mg:41.0 ± 10.4 Eptinezumab 100 mg:41.0 ± 11.7 PLA:39.6 ± 11.3	18 months	a,b,c,d,f,g,j

*PLA* placebo, *a* Adverse Events (AE) and Serious Adverse Events (SAE), *b* monthly migraine days (MMDs), *c* 50% responders rate, *d* 75% responders rate, *e* 100% responders rate, *f* Headache Impact Test (HIT-6) score, *g* headache days, *h* migraine hours, *i* migraines with severe intensity, *j* Percentage of patients with migraine, day 1

### Outcomes

The primary efficacy outcome is mean monthly migraine days (MMDs), baseline to 12 week. Secondary efficacy endpoint included: patients with a 75% reduction in migraine days from baseline (75% responder rate), patients with a 50% reduction in migraine days from baseline (50% responder rate) and patients with migraine 1 day after dosing, baseline to 12 weeks. In addition, we choose the treatment emergent adverse events (TEAEs) as the safety endpoint.

### Summary measures and synthesis of results

Review manager 5.3 was used to assess the data. Estimated standard mean differences and estimated risk ratio (standard mean difference [SMD] or risk ratio [RR]; 95% confidence interval [CI]) were calculated using a random effects model. The I^2^ statistic was used to estimate the statistical heterogeneity as follows: I^2^ < 30% represents “low heterogeneity,” 30% < I^2^ < 50% means “moderate heterogeneity” and I^2^ > 50% means “substantial heterogeneity.” A < 0.05 *P*-value was considered to be significant for all analyses, and tests are two-tailed.

### Risk of Bias

The risk-of-bias plot was assessed using Review Manager 5.3 software (The Cochrane Collaboration, Oxford, UK) for individual studies. The unified standard of the Cochrane Collaboration was applied to assess the risk of bias for RCTs, which included selection bias, performance bias, detection bias, attrition bias, reporting bias, and other potential biases.

## Results

### Search results

**Fig. 1:**
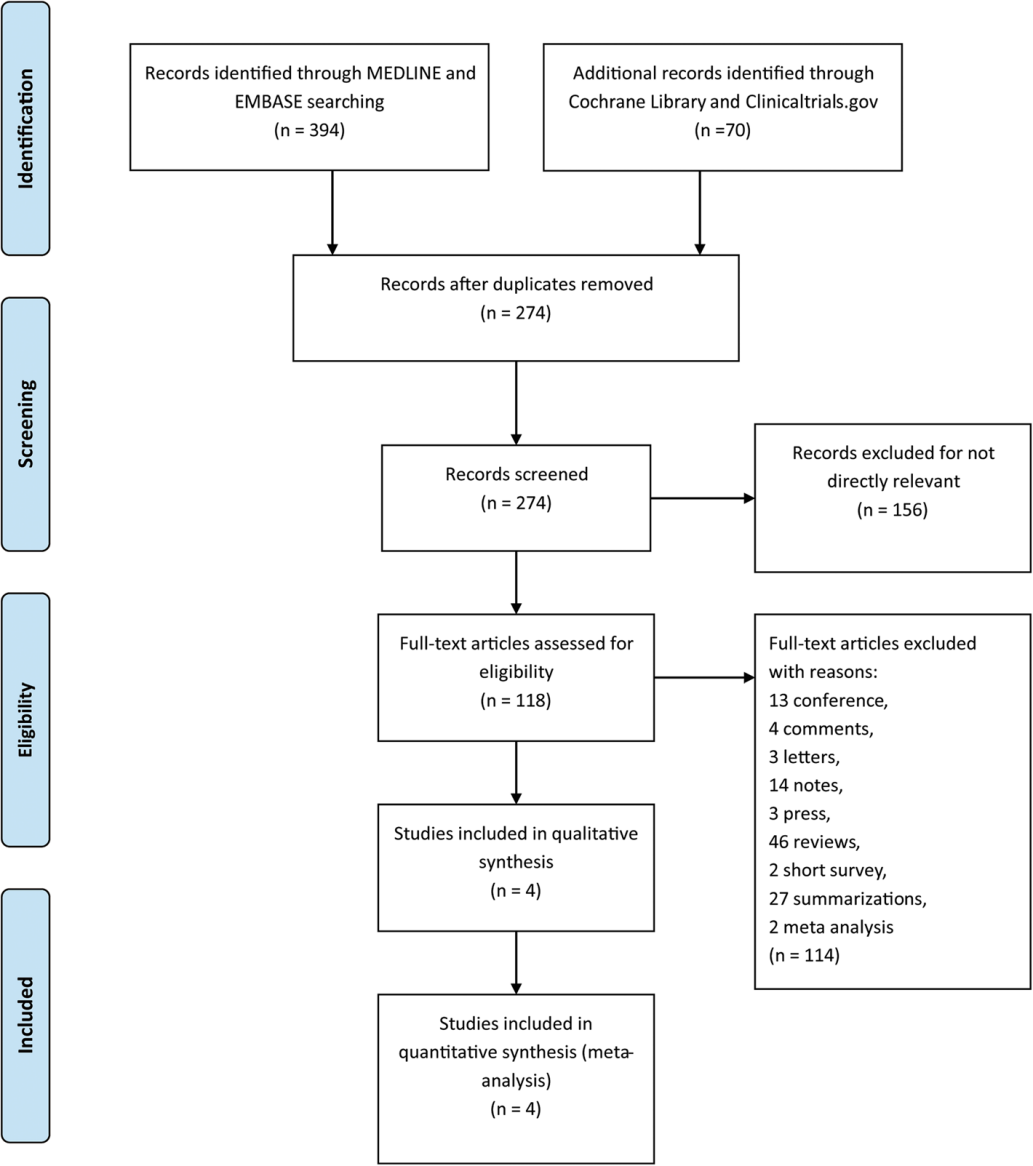
The study search, selection and inclusion process

A total of 464 researches and abstracts from Medline, Embase, Cochrane library and Clinicaltrials.gov were identified. Among them, 190 studies were excluded due to duplicates. Further, 178 studies were excluded as they were irrelevant, such as research on other drugs or into the etiological analysis of migraine. After removing duplicates and uncorrelated titles, 96 of these articles were directly related to the topic of interest. Among them, 92 full text articles were excluded, which included 13 conferences, 4 comments, 46 reviews, 2 short survey and 27 summarizations. Finally, 4 RCTs containing 2739 patients were included in our meta-analysis. The detailed process of screening is shown in Fig. 1.

### Different dosage regimen for the efficacy and safety

**Fig. 2:**
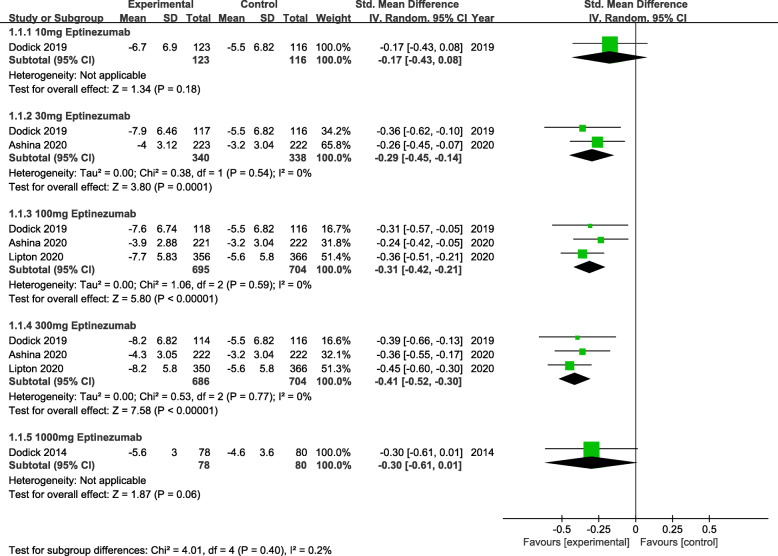
The pooled standard mean difference (SMD) of monthly migraine days (MMDs) in different treatment doses compared with placebo, the diamond indicates the estimated SMD with 95% confidence interval (CI) for the pooled patients

**Fig. 3:**
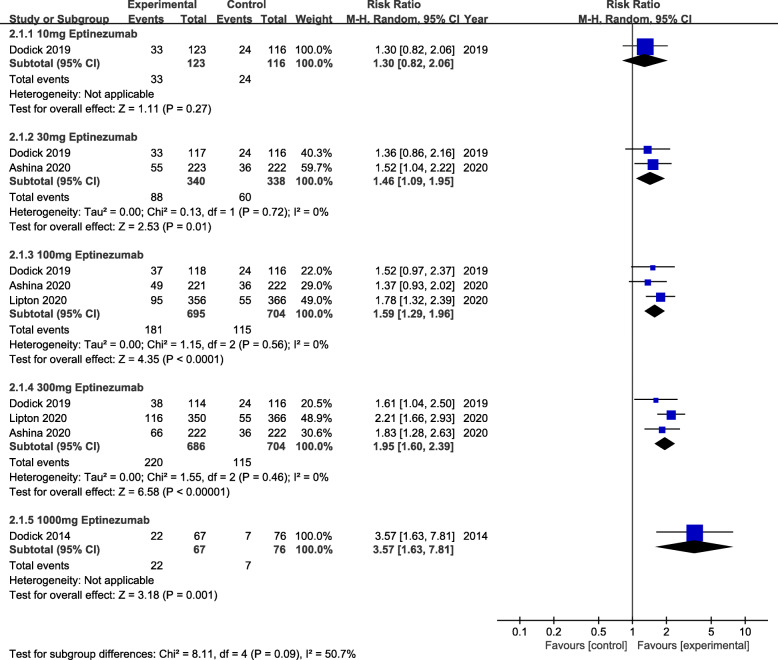
The pooled risk ratio (RR) of 75% responder rate (defined as patients with a 75% reduction in migraine days from baseline) in different treatment doses compared with placebo, the diamond indicates the estimated RR with 95% confidence interval (CI) for the pooled patients

**Fig. 4:**
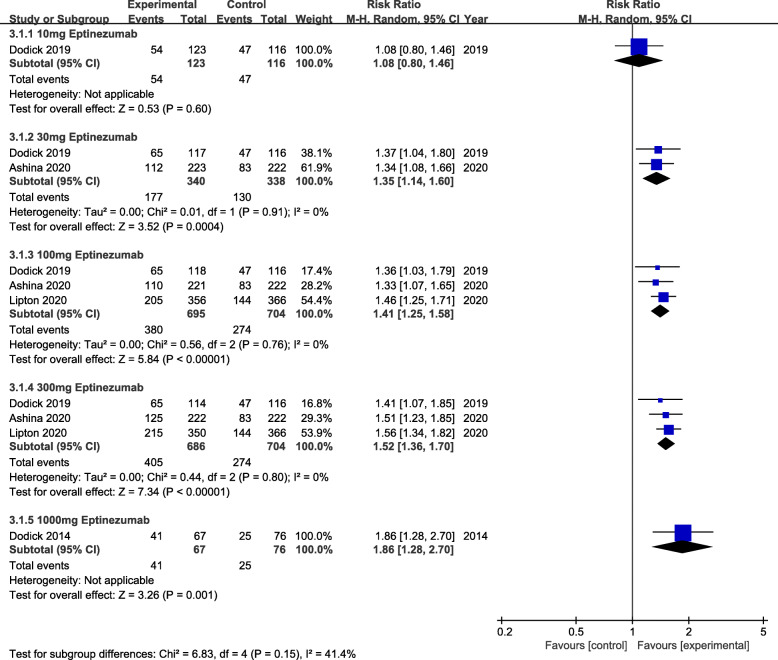
The pooled risk ratio (RR) of 50% responder rate (defined as patients with a 50% reduction in migraine days from baseline) in different treatment doses compared with placebo, the diamond indicates the estimated RR with 95% confidence interval (CI) for the pooled patients

**Fig. 5:**
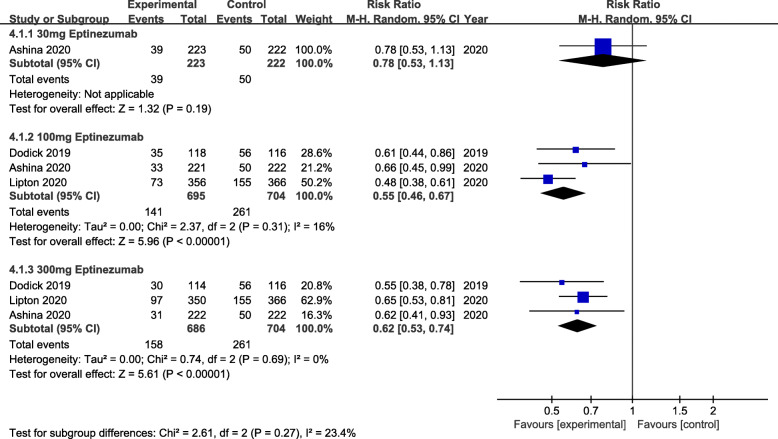
The pooled risk ratio (RR) of patients with migraine 1 day after dosing in different treatment doses compared with placebo, the diamond indicates the estimated RR with 95% confidence interval (CI) for the pooled patients

The primary efficacy outcome was mean monthly migraine day (MMDs) (Fig. 2). From the perspective of primary efficacy outcome, treatment with 30 mg (MD = -0.29, 95% CI:-0.45 ~ − 0.14, *P* = 0.0001), 100 mg (MD = -0.31, 95% CI:-0.42 ~ − 0.21, *P*<0.00001) and 300 mg (MD = -0.41, 95% CI:-0.52 ~ − 0.30, *P*<0.00001) eptinezumab showed significant efficacy compared to the placebo. Whereas, for the secondary efficacy endpoint (75% responder rate, 50% responder rate and patients with migraine 1 day after dosing) (Figs. 3, 4 and 5), the outcomes were abounded with the MMDs. Initially, for the 75% responder rate, treatment with 30 mg(RR = 1.46, 95% CI:1.09 ~ 1.95, *P* = 0.01), 100 mg(RR = 1.59, 95% CI:1.29 ~ 1.96, *P*<0.0001), 300 mg(RR = 1.95, 95% CI:1.60 ~ 2.39, *P*<0.00001) and 1000 mg(RR = 3.57, 95% CI:1.63 ~ 7.81, *P* = 0.001) indicated that they could increase the rate significantly. Further, for the 50% responder rate, treatment with 30 mg (RR = 1.35, 95% CI:1.14 ~ 1.60, *P* = 0.0004), 100 mg (RR = 1.41, 95% CI:1.25 ~ 1.58, *P*<0.00001), 300 mg (RR = 1.52, 95% CI:1.36 ~ 1.70, *P*<0.00001) and 1000 mg (RR = 1.86, 95% CI:1.28 ~ 2.70, *P* = 0.001) eptinezumab also showed improved efficacy compared to the placebo. Nevertheless, from the perspective of the data related to patients with migraine 1 day after dosing, treatment with 100 mg(RR = 0.55, 95% CI:0.46 ~ 0.67, *P*<0.00001) and 300 mg (RR = 0.65, 95% CI:0.53 ~ 0.74, *P*<0.00001) appeared to be more effective than 30 mg(RR = 0.78, 95% CI:0.53 ~ 1.13, *P* = 0.19) compared with the placebo.

**Fig. 6:**
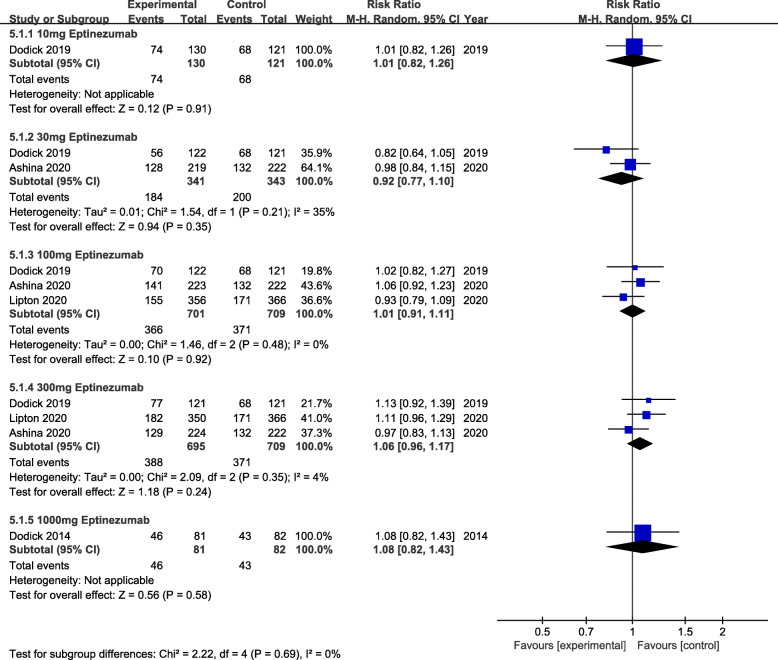
The pooled risk ratio (RR) of patients with treatment emergent adverse events (TEAEs) in different treatment doses compared with placebo, the diamond indicates the estimated RR with 95% confidence interval (CI) for the pooled patients

Of all the 2739 patients receiving eptinezumab, no death occurred during the treatment period. The common adverse effects contained upper respiratory tract infection, nausea and sinus congestion. Therefore, we summarized the adverse events which showed the treatment with 10 mg(RR = 1.01, 95% CI:0.82 ~ 1.26, *p* = 0.91),30 mg(RR = 0.92, 95% CI:0.77 ~ 1.10, *p* = 0.35),100 mg(RR = 1.01, 95% CI:0.91 ~ 1.11, *p* = 0.92), 300 mg(RR = 1.06, 95% CI:0.96 ~ 1.17, *p* = 0.24),1000 mg (RR = 1.08, 95% CI:0.82 ~ 1.43, *p* = 0.58) had no evident statistical difference between eptinezumab and placebo (Fig. 6).

### Dosage regimen of 100 mg vs. 300 mg

**Fig. 7:**
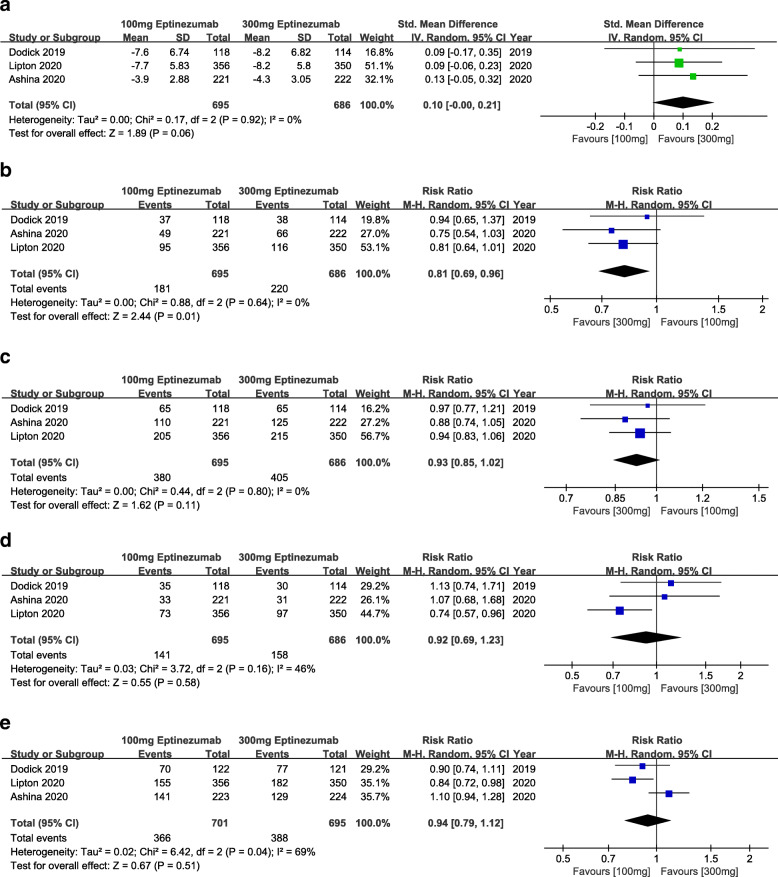
Comparison of efficacy and safety outcomes between 100 mg and 300 mg eptinezumab. a: monthly migraine days (MMDs); b: 75% responder rate; c: 50% responder rate; d: patients with migraine 1 day after dosing; e: treatment emergent adverse events (TEAEs)

Further, study was carried out to compare the efficacy between the 100 mg and 300 mg (Fig. 7a-d). Results from this comparison showed that the treatment with 300 mg(MMDs, MD = 0.10, 95% CI:0.00 ~ 0.21, *p* = 0.06; 75% responder rate, RR = 0.81, 95% CI:0.69 ~ 0.96, *p* = 0.01; 50% responder rate, RR = 0.93, 95% CI:0.85 ~ 1.02, *p* = 0.11; patients with migraine 1 day after dosing, RR = 0.92, 95% CI:0.69 ~ 1.23, *p* = 0.58) was more promising than 100 mg. Meanwhile, as shown in Fig. 7e, no difference existed in TEAEs between the 100 mg and 300 mg(RR = 0.94, 95% CI:0.79 ~ 1.12, *p* = 0.51).

### Risk of bias

**Fig. 8:**
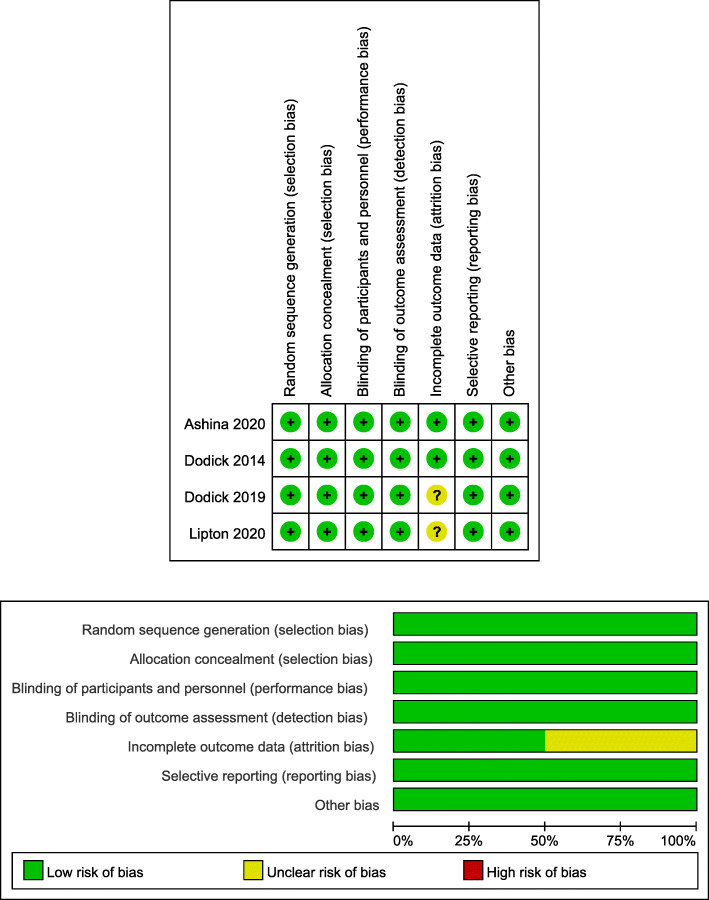
Summary table for potential bias analysis for included study

The independent risk of biases related to 4 RCTs are shown in Fig. 8. The risk for attrition bias is unclear in the studies carried out by Dodick (2019) and Lipton (2020). In addition to the measure, other studies had reported low risks of bias.

## Discussion

Migraine is a prevalent neurological disease around the globe. However, previous therapies have some limitations or adverse effects, and are unresolved until now. As the importance of CGRP in the pathogenesis has been proved by the previous studies, its receptors are widely distributed in the central nervous system (CNS) and peripheral sensory neurons. Therefore, monoclonal antibodies blocking the CGRP ligand or receptor have a clear advantage in the treatment strategy for episodic and chronic migraine [21].

Our study is the first meta-analysis about different dosage regimens related to the safety and efficacy of eptinezumab in the treatment for migraine, and indicated eptinezumab as excellent therapeutic agent for the migraine. Generally, a meta-analysis is a statistical method which combines the results of different researches on the similar topic and it may resolve conflicts among studies. Just as what we have done above, we gathered and analyzed the data from 4 RCTs through objective methods of meta-analysis, which enlarged the sample size and increased statistical power compared with the single available trials. So, this meta-analysis can help clinicians understand eptinezumab in clinical practice and research so that they can conduct a better clinical decision about the use of it.

During our study, we pooled 2739 participators from 4 randomized clinical trials (RCTs), which provided high clinical reliability in the research for the use of eptinezumab. Further, we gathered primary data from these articles and did not discover apparent heterogeneity in our outcomes as indicated by our statistical analysis. Subsequently, we found that eptinezumab had been divided into flexible dosage regimens in these RCTs, including 10 mg, 30 mg, 100 mg, 300 mg, 1000 mg. Further, by comparing the primary efficacy outcomes mean monthly migraine days (MMDs), baseline to 12 weeks, we proved that treatment with 30 mg, 100 mg, 300 mg can cause effective reduction in monthly migraine days (MMDs) compared with placebo. Whereas, for the secondary endpoint, all dosage regimens of eptinezumab increased the proportion of 75% responder rate except 10 mg. Similar results were observed in 50% responder rate. In addition, fewer patients suffered from migraine 1 day after 100 mg and 300 mg eptinezumab administration compared with 30 mg. Due to the lack of research and subsequent data, we could not continue further exploration of 10 mg (only in the study conducted by Dodick et al. 2014) and 1000 mg (only in the study conducted by Dodick et al. 2019) for the efficacy of eptinezumab. However, it doesn’t mean that these dosage regimens were insignificant, probably research related to it needs more time for comprehensive outcome.

By analyzing the results of different dosage regimens of eptinezumab, we found that the dosage regimens of 100 mg and 300 mg were more significant in the efficacy of the treatment for migraine. Moreover, the most recent phase III clinical trial conducted by Lipton et al. 2020 also employed 100 mg and 300 mg eptinezumab, which might confirm the tendency of exploration between the dosage regimens of 100 mg and 300 mg [19]. Therefore, further study was carried out for these two dosage regimens. From the perspective of the outcome related to the MMDs, baseline to 12 week, 300 mg(*P* = 0.06) eptinezumab showed no significant difference but potential tendency for the reduction of MMDs compared with 100 mg. Nevertheless, for the 75% responder rate, 300 mg eptinezumab has been proved more increasing proportion than 100 mg. The result of 50% responder rate and patients with migraine 1 day after dosing couldn’t indicate the difference between 100 mg and 300 mg. To sum up, the dosage regimen of 300 mg may have an advantage on the efficacy of the treatment for migraine. Certainly, the advantage was merely demonstrated by statistical analysis, waiting for more clinical verification.

During our study, the analysis of safety outcomes—TEAEs did not indicate existence of statistical difference between eptinezumab and placebo (*P*>0.05). Therefore, generally the use of eptinezumab is safe for the treatment of migraine. The result was consistent with the meta-analysis conducted by Da Xu and Deng Chen [22] which demonstrated monoclonal antibodies blocking the CGRP ligand or receptor are safe. As reported in the previous studies, we observed that eptinezumab rarely causes serious adverse events or even death [23, 24]. Moreover, it only resulted in some mild adverse events such as upper respiratory tract infection, nausea and sinus congestion, just like the other monoclonal antibodies blocking the CGRP ligand or receptor [25]. Certainly, these studies on adverse events merely evaluated 12 weeks after the first dose. We cannot ensure whether eptinezumab will produce long lasting influence. Therefore, it still needs further comprehensive research.

After the analysis of our data, we found few limitations in our study which cannot be avoided through existing researches. Firstly, numerous previous studies have concluded evidence to use other CGRP monoclonal antibodies such as ubrogepant, galcanezumab and rimegepant, for the treatment of migraine [26–28]. However, as interventions in our study were related to different dosage regimens of eptinezumab and placebo, we can only conclude the advantages of eptinezumab compared with placebo. Probably, our study needs more horizontal comparison of eptinezumab with other CGRP monoclonal antibodies in the future. Secondly, considering different dosage regimens in 4 RCTs, 1000 mg eptinezumab merely conducted by Dodick 2014, whereas, 10 mg merely conducted by Dodick 2019. Moreover, part of statistics from Dodick 2019, Ashina 2020 and Lipton 2020 did not indicate standard deviation (SD) clearly. However, in the present study, we ultimately achieved SD using statistical algorithm on our own. Therefore, the accuracy of the results needs further verification. Thirdly, we combined studies about episodic migraine with those about chronic migraine which may increase the heterogeneity of comparisons. Fourth, this meta-analysis was not registered prior to data collection. Except for the limitation above, we also cannot ignore the lack of adherence in the therapy of migraine which occurred in our 4 RCTs in a way. This also encountered by few traditional treatments for migraine [29, 30].

## Conclusion

In conclusion, eptinezumab showed outstanding efficacy for the treatment of migraine, especially dosage regimen of 300 mg. Meanwhile, no apparent differences existed when compared with placebo from the perspective of safety. Nonetheless, we are looking forward for more studies related to the eptinezumab so that it may have a promising future in the therapy strategy of migraine.

## Supplementary Information

**Additional file 1: Table S1:** Inclusion, exclusion criteria, outcome assessments, conclusions and data acquisition time of the included studies.

## Data Availability

All data generated or analyzed during this study are included in this published article and its supplementary information files.

## Abbreviations

CGRP: Calcitonin gene-related peptide
RCTs: Randomized controlled trials
RR: Risk ratio
SMD: Standard mean difference
MMDs: Monthly migraine day
TEAEs: Treatment emergent adverse events
CI: Confidence interval
CNS: Central nervous system
