# Supplementary material for: Different dosage regimens of Eptinezumab for the treatment of migraine: a meta-analysis from randomized controlled trials
Source: J Headache Pain. 2021 Mar 6;22(1):10. doi: 10.1186/s10194-021-01220-y (PMC7937260; doi:10.1186/s10194-021-01220-y)
Supplement: Supplementary file 1 — Additional file 1: Table S1: Inclusion, exclusion criteria, outcome assessments, conclusions and data acquisition time of the included studies. [file 10194_2021_1220_MOESM1_ESM.docx]

**Different Dosage Regimens of Eptinezumab for the Treatment of Migraine: A Meta-Analysis from Randomized Controlled Trials**

Zeya Yan^1,#^, Tao Xue^1,#^, Shujun Chen^2^, Xin Wu^1^, Xingyu Yang^1^, Guangjie Liu^1^, Shan Gao^3^, Zhouqing Chen ^1^^,^*, Zhong Wang ^1,^*

^1^ *Department of Neurosurgery & Brain and Nerve Research Laboratory,* *The First Affiliated Hospital of Soochow University,* *Suzhou, Jiangsu Province, 215006, China*

^2^ *Department of Neurology, The First Affiliated Hospital of Soochow University, Suzhou, Jiangsu Province, 215006, China*

*^3^ Department of Neurosurgery, The People’s Hospital of SND, Suzhou, Jiangsu Province, 215129, China*

Complete co-author email addresses:

Zeya Yan, E-mail: Dr_Yzeya@163.com

Tao Xue, E-mail: 2992326676@qq.com

Shujun Chen, E-mail: 1026443927@qq.com

Xin Wu, E-mail: 15305130828@163.com

Xingyu Yang, E-mail: yxystarby@163.com

Guangjie Liu, E-mail: [1937247527@qq.com](mailto:1937247527@qq.com)

Shan Gao, E-mail: gaoshan5369@126.com

Zhouqing Chen, E-mail: [zqchen6@163.com](mailto:zqchen6@163.com)

Zhong Wang, E-mail: [wangzhong761@163.com](mailto:wangzhong761@163.com)

^#^ Zeya Yan and Tao Xue contribute equally to this work.

*Corresponding author: Zhouqing Chen or Zhong Wang Department of Neurosurgery, The First Affiliated Hospital of Soochow University, 188 Shizi Street, Suzhou 215006, China. E-mail address: [wangzhong761@163.com](mailto:wangzhong761@163.com) or [zqchen6@163.com](mailto:zqchen6@163.com)

**Table S1: Inclusion, exclusion criteria, outcome assessments, conclusions and data acquisition time of the included studies**

| Trials | Dodick et al 2014  NCT01772524 |
| --- | --- |
| *Inclusion criteria* | 1. Men and women aged between 18 and 55 years with more than 12 months of  migraine who were diagnosed before age 50 years.  2. Patients had an estimated frequency of 5 to 14 migraine days per 28-day period in each of the 3 months before screening and adequately completed an electronic headache diary on at least 25 days of the 28-day screening period. 3. During the 28 days after the screening visit, the patient had to experience at least 5 and up to 14 migraine days as recorded in the electronic diary.  4. Use of acute migraine drugs for 14 days or fewer per 28-day period, including 10 days or fewer of triptan use per 28-day period, in the 3 months before the initial screening visit and during the subsequent 28-day screening period was permitted. |
| *Exclusion*  *criteria* | 1.Participants had a history of regular use (>7 days) of any headache preventive drug or supplement with evidence of efficacy from at least one placebo-controlled trial within 3 months before screening,or received botulinum A toxin within 6 months before screening.  2.Patients with a history of chronic tension-type headache; hypnic headache; hemicrania continua; new daily persistent headache; or basilar-type, sporadic, or  familial hemiplegic migraine were also excluded. |
| *Efficacy outcomes* | 1. The primary efficacy endpoint was the change from baseline to weeks 5–8 in the frequency of migraine days.  2. Other efficacy secondary endpoints: responder rates (patients with a 50% or 75% reduction in migraine days at weeks 1–4, 5–8, and 9–12); mean post-treatment migraine frequencies per month across weeks 1–12; and the change from baseline to weeks 1–4, 5–8, and 9–12 in migraine hours, migraine episodes, frequency of headache days, HIT-6 score, and MSQ score for the subsections role function preventive, role function restrictive, and emotional function.  3. Tertiary endpoints were migraine severity and percentage of migraines with acute migraine treatment. |
| *Safety outcomes* | The most frequent adverse events were upper respiratory tract infection, urinary tract infection, fatigue, back pain, nausea and vommiting, and arthralgia. Most adverse events were transient and mild to moderate in severity. Six serious adverse events were reported by three patients; all of these events were deemed to be unrelated to study drug (appendix). |
| *Conclusions* | There were promising but preliminary safety and efficacy findings for ALD403 in the preventive treatment of migraine. |
| *Data acquisition time* | 1.MMDs, responder rates, migraine hours, migraine episodes, frequency of headache days, HIT-6 score, and MSQ score were measured every 4 months during the study.  2. Adverse events were measured 12 weeks after dose. |

| Trials | Dodick et al 2019  （NCT02275117 |
| --- | --- |
| *Inclusion criteria* | 1. Men and women aged 18–55 years were eligible if they had a diagnosis of CM including a diagnosis of migraine established at age ≤35 years and history of CM of ≥1 year.  2. During the 28-day screening period, patientswere required to have ≥15 headache days, of which ≥8 were assessed as migraine days.  3. Use of hormonal therapy and preventive medications for headache，except botulinum toxin, was allowed if the dosing has been stable for ≥3 months before screening, and was maintained at the same dosing level throughout the trial. |
| *Exclusion*  *criteria* | Multiple protocol violations；Withdrawal. |
| *Efficacy outcomes* | 1. Primary efficacy endpoint ：monthly migraine days;  2. Secondary efficacy endpoint：≥75% migraine responder rates and ≥50% and≥100% reduction in migraine days and headache days; change from baseline in frequency of migraine and headache days; percent change from baseline in migraine and head-ache days; migraine and headache hours; migraines andheadaches with severe intensity; and percentage ofmigraines and headaches with acute medication usage; Headache Impact Test (HIT-6, version 1.0) score. |
| *Safety outcomes* | Safety endpoints included treatment-emergent adverse events (TEAEs) and changes in laboratory parameters,vital signs, 12-lead electrocardiograms, and physical examination findings. |
| *Conclusions* | Results supported the advancement of eptinezumab into phase 3 clinical development to generate additional evidence and confirm its effectiveness for the preventive treatment of episodic and chronic migraine. |
| *Data acquisition time* | 1.MMDs, responder rates were measured at weeks 4, 8, 12, 24,36, and 49 after dosing from the eDiary.  2. HIT-6 scores were measured on day 0 and at weeks 4,12, 24, and 49.  3. Adverse events were measured 12 weeks after dose |

| Trials | Ashina et al 2020  (NCT0255989) |
| --- | --- |
| *Inclusion criteria* | 1.Adults aged 18–75 years (inclusive) with a diagnosis of migraine per ICHD criteria at or before the age of 50 years were eligible for participation if they had a history of migraine for ≥12 months with ≤14 headache days per month, including ≥4 migraine days, in the 3 months prior to screening.  2.Eligible patients were also required to have completed an electronic diary on ≥25 of the 28 days between the screening visit and randomization, documenting ≤14 headache days, including ≥4 migraine days.  3.Patients using acute migraine medications were eligible if use was limited to ≤14 days per 28-day period in the 3 months before screening and during the 28-day screening period; triptan use was limited to ≤10 days per 28-day period in the 3 months prior to screening and the 28-day screening period. Eligible patients could not regularly use (>7days) prophylactic headache medication within 2 months prior to screening and during the 28-day period prior to randomization; short-term (<7 days/month) prophylactic treatment for menstrual migraine was allowed. Patients using barbiturates or prescription opiates ≤4 days/month were eligible if use was stable for ≥2 months prior to screening. Patients using non-prescription codeine preparations containing ≤16 mg codeine were eligible, as well as those using stable hormonal therapy. |
| *Exclusion*  *criteria* | 1.Individuals had confounding pain syndromes or any pain syndrome requiring regular analgesia; uncontrolled or untreated psychiatric conditions; temporomandibular disorders; headache or migraine disorders that did not meet the ICHD-III beta version (2013) section 1.3 criteria for migraine with or without aura; present or previous malignancies or other specified medical conditions.  2.Patients were excluded, who received any experimental unregistered therapy within 30 days or five plasma half-lives before screening; any monoclonal antibody treatment within 6 months of screening; botulinum toxin for any other reason requiring injections in the head, face, or neck within 4 months prior to screening or during the 28-day screening period; or who used approved devices, neuromodulation, neurostimulation, or injectable therapy for headache prophylaxis within 2 months prior to screening or during the 28-day screening period.  3.Individuals were also excluded from participation if they were unable to differentiate migraine from other headaches. |
| *Efficacy outcomes* | Primary outcome: monthly migraine days（MMDs）  Secondary outcomes：75% migraine responder rate，50% migraine responder rate，the percentage of patients with a migraine on the first day after dosing。 |
| *Safety outcomes* | The incidence of TEAEs was generally balanced among treatment groups; no dose-related trends in TEAE incidence were observed. For most patients, these events were mild or moderate. |
| *Conclusion* | Results demonstrate a statistically significant and clinically meaningful migraine preventive effect of eptinezumab in patients with episodic migraine over weeks 1–12 following the first IV administration. Eptinezumab treatment demonstrated acceptable safety and tolerability across doses compared to placebo, with no apparent dose-related trend in the nature, frequency, or severity of TEAEs. The percentage of patients with anyTEAE was similar across the eptinezumab and placebo groups, with most events being mild or moderate in severity. |
| *Data acquisition time* | MMDs，75% migraine responder rate and 50% migraine responder rate were measured by the eDiary at 4, 8 and 12 weeks after randomization. |

| Trials | Lipton et al 2020  (NCT02974153) |
| --- | --- |
| *Inclusion criteria* | Adults 18 to 65 years of age (inclusive) with a diagnosis of migraine at or before 50 years of age were eligible for participation if they had a history of CM for ≥12 months before screening, completed the headache electronic diary (eDiary) on≥24 of the 28 days after screening visit and before randomization (the screening period), and experienced ≥15 to ≤26 headache days and ≥8 migraine days during the 28-day screening period.2Patients taking prescription or over-the-counter medication for acute or preventive treatment of migraine were eligible only if the medications had been prescribed or recommended by a health care professional; migraine preventive medication use had to be stable for ≥3months before screening. Hormonal therapy was also permitted if it was stable and ongoing≥3months before screening. Patients using barbiturates or prescription opioids ≤4 d/o were eligible for participation if use was stable for ≥2 months before screening, and this restriction was maintained through week 24 of the study. Other medications for the treatment of acute migraine such as triptans,nonsteroidal anti-inflammatory drugs, and simple analgesics were not restricted. Patients with CM and medication-overuse headache with the exception of the overuse of barbiturates or opioids were eligible for inclusion. |
| *Exclusion*  *criteria* | 1.patients using opioids or barbiturates ≥5 d/mo were excluded.  2. Individuals were excluded from participation if they had a confounding pain disorder or clinically significant pain syndromes;uncontrolled or untreated psychiatric conditions; acute or active temporomandibular disorders; history or diagnosis of a headache or migraine disorders that did not meet the International Classification of Headache Disorders, 3rd edition (ICHD-3) beta version (2013) Section 1.3 criteria for CM; present or previous malignancies (except history of squamous or basal cell carcinoma with excision for cure or breast or cervical cancer≥10 years since diagnosis/treatment without evidence of recurrence); any active, progressive, or unstable cardiovascular, neurologic, or autoimmune disorder; newly diagnosed or uncontrolled hypertension (mild primary hypertension that was well controlled for ≥6 months before screening was allowed);history or evidence of substance abuse or dependence; clinically significant abnormal ECG findings; a concurrent medical condition or laboratory abnormality during the screening period or before dosing on day 0; body mass index ≥39 kg/m^2^; or recent or planned surgery requiring general anesthesia within 8 weeks before screening or during the duration of the study. Also excluded were patients who had received any experimental, unregistered therapy within 30 days or 5 plasma half-lives before screening or who had used any prohibited devices, neuromodulation, neurostimulation, or injectable therapy within 2 months before screening or during the screening period; botulinum toxin (any type) for migraine or for any other medical/cosmetic reasons requiring injections within 4 months before screening or during the screening period; monoamine oxidase inhibitors, ketamine, methylergonovine, or nimesulide within 3 months before screening or during the screening period; any monoclonal antibody treatment within 6 months of screening; or eptinezumab or any monoclonal antibody targeting the CGRP pathway. Women who were pregnant, breastfeeding, or planning to become pregnant during the study were excluded from participation, as were patients participating in any other clinical study or who were positive for HIV, hepatitis B surface antigen, or hepatitis C. |
| *Efficacy outcomes* | Primary efficacy endpoint: monthly migraine days (MMDs)  Key secondary efficacy endpoints: The ≥75% migraine responder rates, ≥50% migraine responder rates, mean headache days, percentage of patients with migraine on day 1, mean acute medication days and HIT-6 score. |
| *Safety outcomes* | Overall, 508 patients (47.4%) experienced ≥1 treatment-emergent AEs (TEAEs). The incidence of TEAEs was generally balanced among treatment groups. The most frequently reported study drug-related TEAEs were fatigue. No deaths were reported in this study. |
| *Conclusion* | Results demonstrated that both the 100 and 300 mg doses of eptinezumab resulted in significant reductions in the primary endpoint, MMDs, over weeks 1 to 12 after the first IV administration. The ≥75% and ≥50% migraine responder rates for the eptinezumab 100 mg and 300 mg dose groups were greater than for placebo. These findings are consistent with previous results from the phase 2 study of CM. |
| *Data acquisition time* | Patients completed a daily eDiary from the time of screening through week 24; this included a daily evening report (completed regardless of whether the patient had a headache) and a headache report, which was event based. Monthly results were based on the results of the 4-week intervals. |
